# Automatic activation of neurostimulation for central sleep apnea results in high nightly usage

**DOI:** 10.1007/s44470-026-00051-5

**Published:** 2026-02-27

**Authors:** Rami Khayat, Meena Khan, Timothy I. Morgenthaler, Scott McKane, Robin Germany, Maria Rosa Costanzo

**Affiliations:** 1 Pulmonary, Allergy, Critical Care, and Sleep Medicine, Penn State University, Hershey, PA USA; 2 Division of Pulmonary Critical Care and Sleep Medicine, Department of Internal Medicine, Ohio State University Medical Center, Columbus, OH USA; 3 Center for Sleep Medicine, Division of Pulmonary and Critical Care Medicine, Mayo Clinic, Rochester, MN USA; 4 ZOLL Respicardia, Inc., Minnetonka, MN USA; 5 Midwest Cardiovascular Institute, Naperville, IL USA

**Keywords:** Central sleep apnea, Adherence, Transvenous phrenic nerve stimulation

## Abstract

**Purpose:**

Adherence with mask-based therapies remains a significant challenge for patients with sleep-disordered breathing. Transvenous phrenic nerve stimulation (TPNS) to treat central sleep apnea (CSA) in adults offers a novel, automated approach to enhance nightly adherence by initiating therapy automatically based on patient-specific programmed conditions such as sleep schedule, activity, and body position. This retrospective analysis aims to determine the adherence achieved with TPNS’s automated activation approach.

**Methods:**

Device data from 131 remedē^®^System Pivotal Trial participants were analyzed to calculate median daily hours of therapy within 14 days prior to visits. Therapy duration was defined as time when all conditions to activate therapy were met between the first-time therapy on to last time off during the night.

**Results:**

Median [Q1-Q3] nightly therapy duration at 6, 12, 18, and 24 months was 5.9 [4.9–6.6], 5.7 [5.0-6.8], 6.0 [5.3–6.8], and 5.9 [4.9–6.5] hours, respectively. Using a definition for adequate therapy as the percentage of patients with usage ≥ 4 h/night for 70% of nights, 86%, 82%, 83%, and 90% of patients met the criteria at these visits. Two patients (< 2%) discontinued treatment before 6 months for stimulation intolerance. Patients with adequate therapy reported more quality of life improvement.

**Conclusion:**

Automatic activation of TPNS therapy was associated with consistent use in over 80% of patients through 18 months. Higher usage may yield a greater overall reduction in CSA burden over the whole night compared to therapies requiring the patient to initiate the therapy.

**ClinicalTrials.gov identifier:**

NCT01816776 (21MAR2013)

**Brief summary:**

**Current Knowledge/Study Rationale**: Adherence to mask-based therapies has been a challenge. Usage and adherence of transvenous phrenic nerve stimulation therapy has a potential to be higher with automatic therapy activation when programmed conditions are met.

**Study Impact: **The automatic activation of transvenous phrenic nerve stimulation therapy resulted in a higher duration of nightly usage and higher rate of adequate delivered therapy than generally are reported for mask-based therapies. High usage may yield a greater overall reduction in central sleep apnea burden over the whole night compared to therapies that require the patient to initiate the therapy, especially later in the night when CSA is more likely to occur.

## Introduction

Central sleep apnea (CSA) is a type of sleep-disordered breathing (SDB) that is most encountered in patients with cardiovascular disease [1–3]. The underlying mechanism of CSA is respiratory control instability that encompasses a spectrum of disorders ranging from Cheyne-Stokes Respiration, found mainly in patients with heart failure, to a transient form of CSA associated with high altitude. Other syndromes include opioid associated CSA and treatment-emergent CSA. The American Academy of Sleep Medicine recently published updated guidelines for the treatment of CSA [4]. Positive airway pressure (PAP) devices including continuous positive airway pressure (CPAP) and adaptive servo ventilation devices continue to play a large role in the treatment of CSA according to these guidelines. However, adherence to mask-based therapies remains a significant challenge in patients with SDB. For example, adherence to CPAP devices was reported between 2.7 and 3.3 h per night on recent randomized controlled trials for obstructive sleep apnea (OSA) [5, 6]. The Center for Medicare & Medicaid Services (CMS) requires that patients use their PAP devices for at least four hours per night for 70% of nights which is a relatively low target yet is difficult for many patients to meet [7]. A recent analysis supported that a minimum usage threshold of 4- hour may be important for patients with OSA to experience the cardiovascular risk reduction [8]. Many patients stop using PAP devices or remove the mask due to comfort and tolerability issues, and adherence has been shown to be related to age and sex [9]. Similar rates of adherence have been seen in studies with CSA utilizing CPAP or more advanced forms of PAP therapy [10, 11].

Neurostimulation devices for obstructive sleep apnea appear to have a better adherence rate than mask therapies, with last follow-up in the ADHERE database (1–2 years) showing 82% of patients using the therapy > 4 h per night [12].

**Fig. 1:**
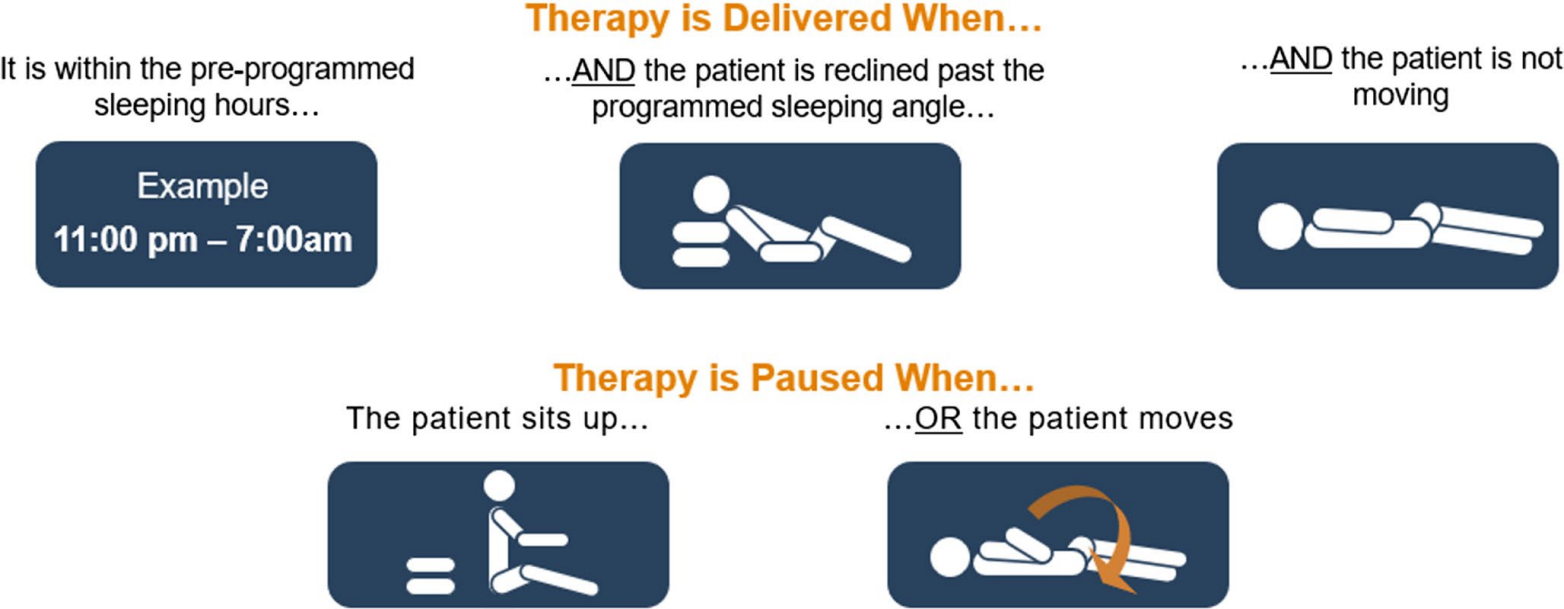
Therapy activation requirements. Therapy activates automatically each night based on programmed schedule and accelerometer readings. Therapy pauses if the patient recline position is greater than the programmed angle, if the patient activity is above the programmed threshold, or if the patient roles over. Therapy resumes ~ 10 min (programmable duration) after the patient returns to a reclined position and is once again still. (Courtesy of ZOLL Respicardia, Inc.)

Transvenous phrenic nerve stimulation (TPNS) treats moderate to severe CSA in adult patients. TPNS offers a novel, automated approach to enhance nightly adherence by initiating therapy based on patient-specific programmed conditions such as sleep schedule, activity, and body position. Therapy automatically pauses when the patient sits up or changes position, facilitating re-initiation of sleep before therapy resumes (Fig. 1). It is expected that this automatic delivery may be helpful to ensure use of this therapy in CSA. TPNS therapy demonstrates effectiveness in the management of CSA, showing improvements in respiratory and sleep parameters, and overall quality of life [13, 14]. The modality was approved by the Food and Drug Administration in 2017 for the treatment of CSA in adults and was recently added to the AASM Central Sleep Apnea Guidelines as a treatment option clinicians should offer to most patients if clinically appropriate [4].

This retrospective analysis aimed to determine the degree of adherence achieved with TPNS’s automated activation approach. The impact of this approach to automatic delivery of therapy on overall treatment duration has not been previously evaluated.

## Methods

### Device

The TPNS device consists of a stimulation lead placed in the brachiocephalic or pericardiophrenic vein, which run adjacent to the phrenic nerve, and an implantable pulse generator implanted by an electrophysiologist [15]. The implantable pulse generator contains an accelerometer that measures patient activity, pitch (or angle), and position (sleep quadrant). Therapy activates automatically each night based on programmed schedule and accelerometer readings. The device activates automatically during the programmed sleep time when the patient is reclined below their programmed sleeping angle (i.e., pitch), and activity level is below the set activity threshold to indicate the patient is at rest (Fig. 1). Therapy pauses if the patient recline position is greater than the programmed angle, if the patient is active, or if the patient turns to a different axial position. Therapy resumes ~ 10 min (programmable duration) after the patient returns to a reclined position and is once again still.

### Population

Subjects from the randomized remedē^®^ System Pivotal Trial (ClinicalTrials.gov identifier: NCT01816776) were included in this retrospective analysis. The protocol was approved by local ethics or institutional review boards and all subjects provided written informed consent to participate in the study. The investigation was performed in accordance with the principles outlined in the Declaration of Helsinki. All subjects were receiving optimal medical management for any comorbidities. In the treatment group, stimulation therapy was activated about 1 month after device implantation to allow for lead stabilization and healing. In the control group, the device was implanted but therapy remained off until after the 6-month post–therapy initiation assessments, at which time stimulation was activated, marking the end of the randomized phase of the trial [13, 14].

In the trial, participants completed an in-laboratory, attended polysomnogram at baseline to determine moderate to severe CSA eligibility (i.e., apnea-hypopnea index ≥ 20 and other criteria to ensure the apneas were primarily central in origin). At the 6-, 12-, and 18-Month visits, subjects completed polysomnograms. Other assessments, including the Patient Global Assessment (PGA) and Epworth Sleepiness Scale (ESS), were completed after 6 and 12 months of active therapy. The PGA was a 7-category response scale that asked “Specifically in reference to your overall health, how do you feel today as compared to how you felt before having your device implanted”, with response choices of markedly improved, moderately improved, mildly improved, no change, slightly worse, mildly worse, and markedly worse.

### Statistical methods

**Fig. 2:**
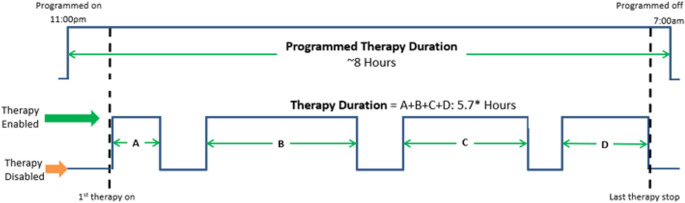
Definition of therapy duration. The figure shows the programmed time on (11:00pm) and off (7:00am), called programmed therapy duration. Therapy duration is the time of delivered therapy when all conditions to activate therapy are met between the 1st time on and last time off during the night (A + B + C + D). Time when programmed criteria for therapy activation is not met was excluded from the therapy duration calculation

A retrospective analysis of device data from participants in the remedē^®^ System Pivotal Trial was performed to quantify nightly therapy use. At each scheduled study visit (6, 12, 18, and 24 months), device data were routinely downloaded at check-in, typically providing 14 consecutive days of therapy information preceding the visit. These 14-day data intervals were used to calculate each subject’s median nightly therapy duration, followed by calculation of overall group medians [first quartile, third quartile]. Therapy duration was defined as the total time stimulation was delivered when all programmed activation conditions were met (Fig. 2).

The randomized groups were pooled for analysis based on months since therapy activation. Additional analyses assessed (1) the median duration of therapy per night achieved and (2) adherence, defined as therapy use ≥ 4 h per night for at least 70% of nights. We also performed an exploratory assessment of the association of these metrics of adherence and quality of life measures.

For these calculations, nights with unavailable device data were excluded. Nights without therapy delivery were assigned 0 h of use when the absence of therapy was attributable to discomfort or programming issues. At the 6-month visit, zero values were assigned for two participants—one who suppressed therapy for three nights using a magnet after an optimization visit, and one temporarily programmed to monitor mode for three nights due to discomfort. Zero values were also assigned for two additional participants with incorrectly programmed sleep windows before the 6- and 12-month visits, respectively; both exhibited normal use at adjacent visits. Other missing nights, such as those without a device file or with incomplete 14-day data coverage, were excluded from analysis.

## Results

Device data was available for 131 subjects (of the 137 with therapy activated) including 128 subjects at 6 months of active therapy, 114 at 12 months, and 103 at 18 months. The reasons for missing data at 6 months were missed visits (*n* = 3), exited study prior to visit (*n* = 2), and device data not collected (*n* = 4); reasons for missing data for later time points were similar. Baseline characteristics of participants are displayed in Table 1.

**Table 1:** Baseline characteristics

Characteristic	Subjects with therapy activated (*N* = 137)
Age (years)	66 [59, 74]
Body mass index (kg/m^2^)	30 [27, 35]
Male sex	90% (123)
White race	96% (131)
Ethnicity not Hispanic or Latino	96% (132)
Heart failure	64% (87/137)
Atrial fibrillation	42% (57/137)
Apnea-hypopnea index (events/hour)	44 [32, 58]
Central apnea index (events/hour)	23 [14, 40]
Obstructive apnea index (events/hour)	2 [< 1, 3]

Categorical reported as percent (n). Continuous reported as median [1st, 3rd quartile]

**Fig. 3:**
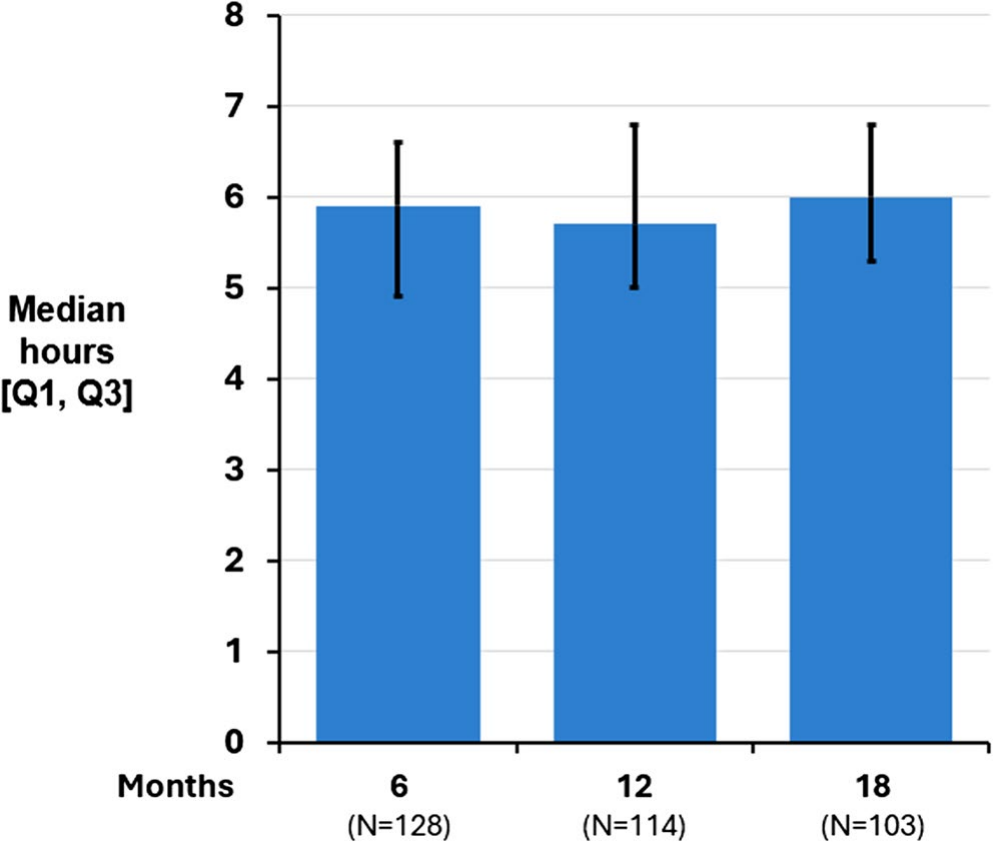
Median nightly therapy duration. Median nightly therapy duration was consistently high at each visit through 18 months at approximately 6 h per night. Only 2 patients (< 2%) discontinued treatment prior to 6 months due to stimulation intolerance

The median [Q1, Q3] nightly therapy duration was 5.9 [4.9, 6.6] hours at 6 months, 5.7 [5.0, 6.8] hours at 12 months, and 6.0 [5.3, 6.8] hours at 18 months (Fig. 3). Subgroup analysis demonstrated therapy duration was similar for those with and without heart failure: the heart failure subgroup had median of 5.9, 5.8, and 6.0 h of use at 6, 12, and 18 months, respectively, compared to 5.8, 5.7, and 6.0 h for the subgroup without heart failure. Due to trial closure following regulatory approval of the device in the United States, a smaller subgroup of subjects (*N* = 42) had data available at 24 months of therapy and that showed a similar duration of 5.9 [4.9, 6.5] hours.

Dividing therapy duration into hours of use per night for the 128 subjects at the 6-month visit, 19% (24 subjects) had ≥ 7 h, 26% (33) with 6-<7 h, 28% (36) with 5-<6 h, 18% (23) with 4-<5 h, and only 9% (12) with < 4 h. This distribution remained similar at each follow-up visit as displayed in Table 2.

**Table 2:** Hours of therapy duration by visit

Months of active therapy	< 4 h	4-<5 h	5-<6 h	6-<7 h	≥ 7 h
6 months (*N* = 128)	9% (12)	18% (23)	28% (36)	26% (33)	19% (24)
12 months (*N* = 114)	11% (12)	13% (15)	36% (41)	23% (26)	18% (20)
18 months (*N* = 103)	11% (11)	11% (11)	30% (31)	28% (29)	20% (103)

Reported as percent (n)

**Fig. 4:**
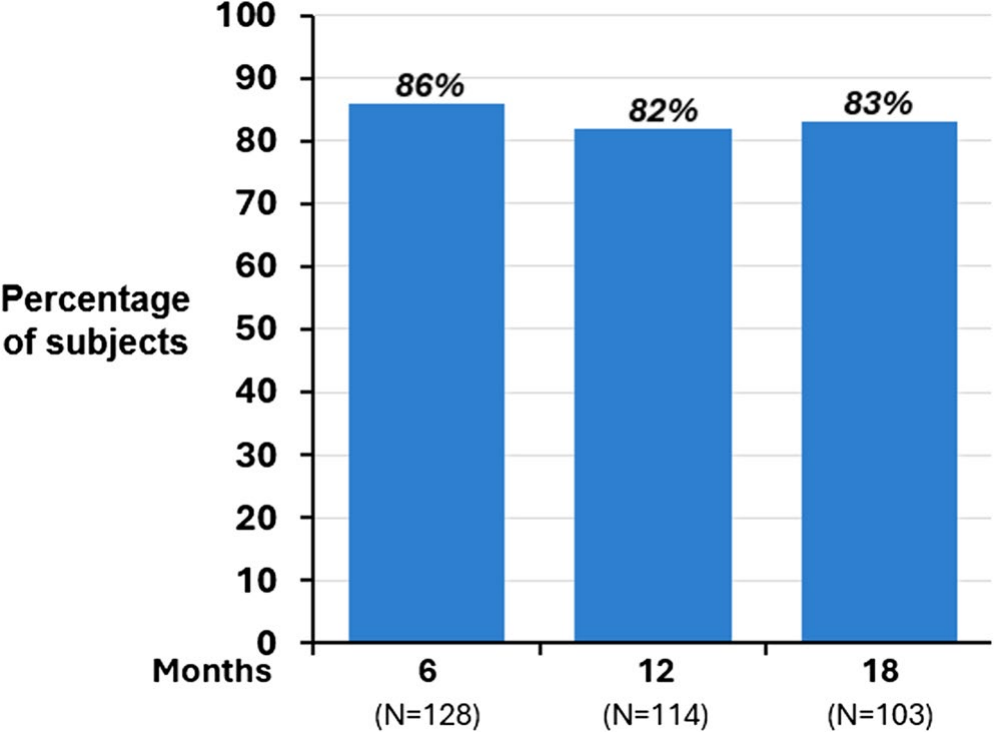
Adequate therapy by visit. Adequate therapy was defined as ≥ 4 h/night for 70% of nights

Using a definition for adequate therapy as the percentage of patients with usage ≥ 4 h/night for at least 70% of nights, 86% (110/128), 82% (94/114), and 83% (85/103) of patients met the criteria at the 6-, 12- and 18-month visits, respectively (Fig. 4). Considering all possible therapy nights in the 14-day period prior to each visit independent of the participant, the percentage of nights with ≥ 4 h of usage was 85% (1,524 out of possible 1,792 patient nights), 86% (1,363/1,593 patient nights), and 87% (1,231/1,418 patient nights) at the 6, 12, and 18 months of therapy, respectively.

Only two patients (< 2%) who had therapy activated discontinued treatment prior to 6 months due to stimulation intolerance. In contrast, after 6 months of active therapy patients were asked if they would elect to have this device implanted again and 95% of the patients responded “Yes”.

**Fig. 5:**
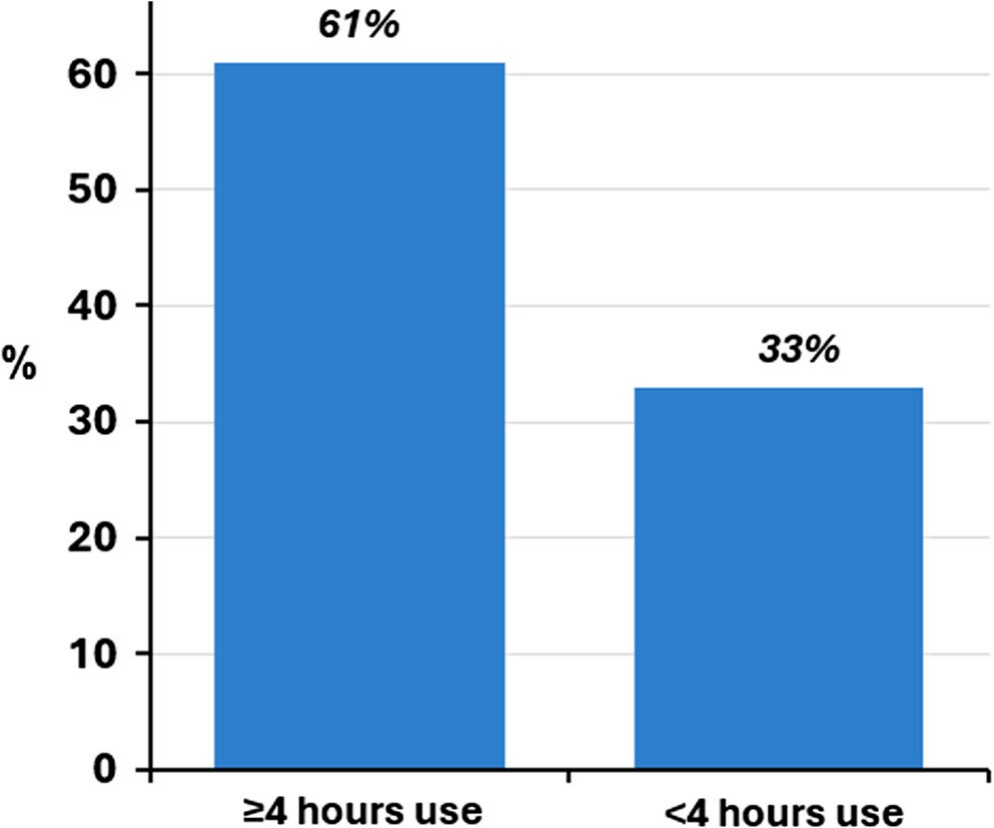
Percent of subjects with marked or moderate improvement on patient global assessment. Patients with ≥ 4 h of therapy per night for 70% of nights experienced a higher percentage of subjects with marked or moderate improvement on the Patient Global Assessment at 6 months

An exploratory analysis of patient reported outcomes was performed to attempt to identify a minimum effective dose that may convey more benefit to patients. Patients with median delivered therapy ≥ 4 h/night reported more quality of life improvement than patients with < 4 h of therapy, as demonstrated by 61% (71/116 subjects) with ≥ 4 h use indicating marked or moderate improvement per the PGA compared to 33% (4/12) of subjects with < 4 h use (Fig. 5). A subsequent anlaysis to assess the impact of each hour of additional therapy duration demonstrated the highest improvement rate (75% [27/36]) in the 5-<6 h range, however response rates did not continue to increase for the 6 or ≥ 7 h use groups.

Daytime sleepiness based on ESS improved incrementally more at 6 months in subjects with ≥ 4 versus < 4 h use, with − 2.5 [-6.0, 0] point change versus − 2.0 [-5.5, − 0.5] points, respectively. After 12 months, the ≥ 4 h use group showed a larger difference: -3.0 [-7.0, 0] versus − 1.5 [-7.5, 0]. Additional analysis again showed that 5–6 h use may result in slightly better improvements, however, improvements were also not consistent in showing incremental improvement with more use beyond 5–6 h.

For subjects with < 4 h of therapy duration, 83% (10/12) indicated they would have the device implanted again compared to 97% (111/115) of those with ≥ 4 h of therapy duration. Analyzed by hour of use, the results were similarly high for each hour over 4 h of use.

## Discussion

The novel automated therapy activation of TPNS resulted in a high level of adherence of approximately 6 h per night and was maintained long term. The percentage of patients receiving adequate therapy, based on the CMS CPAP adherence definition, appears to be higher than that for mask-based therapies reported in the literature. The high level of usage may be associated with the novel automated therapy delivery approach as well as with patient acceptance of the device, with 95% indicating they would undergo the implant of this device again.

Exploratory analysis demonstrated that longer therapy duration may be associated with a positive impact on quality of life and daytime sleepiness. Patients with < 4 h of therapy duration did not experience as much benefit according to the PGA or ESS as those with four or more hours. Exploratory analysis of the hours of use beyond four hours suggests potential for additional benefit with more hours of use, however there was not a consistent relationship of more use with better outcome. Future analysis with larger sample size is needed to confirm the whether there is a minimum effective dose to impact patient reported outcomes.

Maximizing therapy duration is important since it has been shown that CSA worsens later in the night [16]. Therefore, if adherence is low and patients stop using a therapy part way through the night, they experience significant CSA without receiving appropriate treatment when it is needed most. Treatment of CSA requires a multidisciplinary approach and access to expertise in advanced therapies as recently noted by the AASM guidelines on the treatment of CSA (ref). Participants in this trial received structured follow-up and monitoring, which may enhance adherence compared with real-world practice. However, current standards recommend regular follow up and evaluation of efficacy of treatment.

Automated therapy delivery has not been used as part of sleep therapies prior to the TPNS system. Automatic onset and offset of therapy were designed to correspond with awakenings and changing in position to allow for smooth transition into sleep. CSA, however, may immediately start at sleep onset, and the latency to therapy start and slope of the ramp must be programmed to ensure effective therapy reinitiation [16]. Since patients may experience CSA at sleep onset, resumption of therapy delivery should be fast while still allowing the patient to fall asleep prior to full therapy resumption. Various programming features and customizations are possible based on patient preference, ability to fall asleep with the device active, reported sleep pattern, and preferred sleep positions.

The high therapy time observed with TPNS in this study may, in part, be attributed to the automated nature of therapy delivery. Unlike PAP therapy, which requires nightly patient initiation and mask application, this system activates automatically when programmed physiologic conditions are met, thereby removing the burden of engagement from the patient at the time of use.

Similar patterns of improved adherence and outcomes have been demonstrated in other therapeutic areas where automation or clinician-controlled initiation minimizes the burden of daily decision-making. For example, automated insulin delivery systems for diabetes management have consistently improved glycemic control while alleviating diabetes-related distress and improving quality of life [17–19] and long-acting injectable antipsychotics achieve significantly higher adherence compared with oral formulations, reducing relapse and hospitalization rates despite higher drug costs [20–22]. Therefore, therapies that automate delivery or simplify activation tend to improve treatment adherence and, often, clinical outcomes. Automated TPNS appears to fit within this paradigm—providing a durable, low-burden approach that may help sustain long-term use in patients with CSA.

This retrospective analysis had several limitations. First, analysis of device-recorded data from a clinical trial not originally designed to evaluate adherence mechanisms, and therefore residual confounding cannot be excluded. Second, nights without data could arise from technical or download issues, and although these were excluded or assigned to be zero using predefined rules, some bias may remain; Similarly, while we do not see evidence of additional patients discontinuing the study or missing assessments due to tolerability issues beyond the two mentioned, it is a possibility. Third, participants in the trial received structured follow-up and monitoring, which may enhance adherence compared with real-world practice. Fourth, quality-of-life associations were exploratory and cannot establish causality. Fifth, given the high level of adherence and small sample size with low hours use, assessment of a minimal effective dose to achieve quality of life benefits was unable to be fully examined and should be considered exploratory. Last, ability to directly compare use to other CSA or OSA therapies was also unable to be performed since all subjects in this study were using TPNS and therefor comparisons to use of other devices for CSA or OSA is not truly comparative and should be interpreted with caution.

## Conclusion

The automatic activation of TPNS therapy resulted in a higher duration of nightly usage and higher rate of adequate delivered therapy than generally are reported for mask-based therapies. This high usage may yield a greater overall reduction in central sleep apnea burden over the whole night and translate to better outcomes compared to therapies that require the patient to initiate the therapy and stay compliant throughout the night.

## Data Availability

Data is available upon reasonable request.

## Declarations

### Clinical trial

This is a report on the Pivotal Trial of the remedē System, ClinicalTrials.gov identifier: NCT01816776 (21MAR2013).

## Abbreviations

CMS: Center for Medicare & Medicaid Services
CPAP: Continuous positive airway pressure
CSA: Central sleep apnea
ESS: Epworth Sleepiness Scale
OSA: Obstructive sleep apnea
PAP: Positive airway pressure
PGA: Patient Global Assessment
SDB: Sleep-disordered breathing
TPNS: Transvenous phrenic nerve stimulation
